# A digital tool for multidimensional assessment and prediction of treatment effectiveness in chronic pain management

**DOI:** 10.1016/j.isci.2024.111200

**Published:** 2024-10-18

**Authors:** Philippe Rigoard, Amine Ounajim, Maarten Moens, Lisa Goudman, Manuel Roulaud, Nicolas Naiditch, Raouf Boukenna, Philippe Page, Bénédicte Bouche, Bertille Lorgeoux, Sandrine Baron, Kevin Nivole, Mathilde Many, Lucie Lampert, Géraldine Brumauld de Montgazon, Brigitte Roy-Moreau, Romain David, Maxime Billot

**Affiliations:** 1 CHU de Poitiers, PRISMATICS Lab (Predictive Research in Spine/Neuromodulation Management and Thoracic Innovation/Cardiac Surgery), 86000 Poitiers, France; 2 CHU de Poitiers, service de neurochirurgie du rachis, chirurgie de la douleur et du handicap, 86000 Poitiers, France; 3 Université de Poitiers, Pprime Institute UPR 3346, CNRS, ISAE-ENSMA, 86000 Poitiers, France; 4 Department of Neurosurgery, Universitair Ziekenhuis Brussel, 1090 Brussels, Belgium; 5 STIMULUS consortium (reSearch and TeachIng neuroModULation Uz bruSsel), Vrije Universiteit Brussel, Laarbeeklaan 103, 1090 Brussels, Belgium; 6 Department of Radiology, Universitair Ziekenhuis Brussel, Laarbeeklaan 101, 1090 Brussels, Belgium; 7 Research Foundation—Flanders (FWO), 1090 Brussels, Belgium; 8 Pain Evaluation and Treatment Centre, La Rochelle Hospital, 17000 La Rochelle, France; 9 Pain Evaluation and Treatment Centre, Nord Deux-Sèvres Hospital, 79350 Faye-l'Abbesse, France; 10 CHU de Poitiers, service de médecine physique et réadaptation, 86000 Poitiers, France; 11 Centre de Recherche sur la Cognition et l’Apprentissage, Université de Poitiers, Université François Rabelais de Tours, CNRS, 86000 Poitiers, France

**Keywords:** Public health, Pain management in health technology

## Abstract

Given the multidimensional aspect of pain, the assessment of treatment efficacy is challenging. The prospective observational multicenter PREDIBACK study aimed to assess, compare, and predict the effectiveness of different treatments for persistent spinal pain syndrome type 2 (PSPS-T2) using a digital tool and the Multidimensional Clinical Response Index (MCRI) including pain intensity, functional disability, quality of life, anxiety and depression, and pain surface. Results indicated that neurostimulation was the most effective treatment at 3-, 6-, 9-, and 12-month follow-up compared to baseline, leading to significant improvements in pain, function, and quality of life, whereas optimized medical management (OMM) and spinal reoperation showed no significant benefits. Additionally, the study identified pain surface, BMI, and smoking status as predictors of treatment outcomes. These findings highlight the potential of digital medicine to improve patient care by providing data-driven insights and personalized treatment recommendations for PSPS-T2.

## Highlights

• Digital tool captures multidimensional clinical response in chronic pain patients
• Neurostimulation shows significant improvement over OMM and reoperation
• Pain surface, BMI, and smoking are predictors of treatment outcomes
• Digital medicine offers potential for personalized treatment decisions

## Graphical abstract

**Figure:**
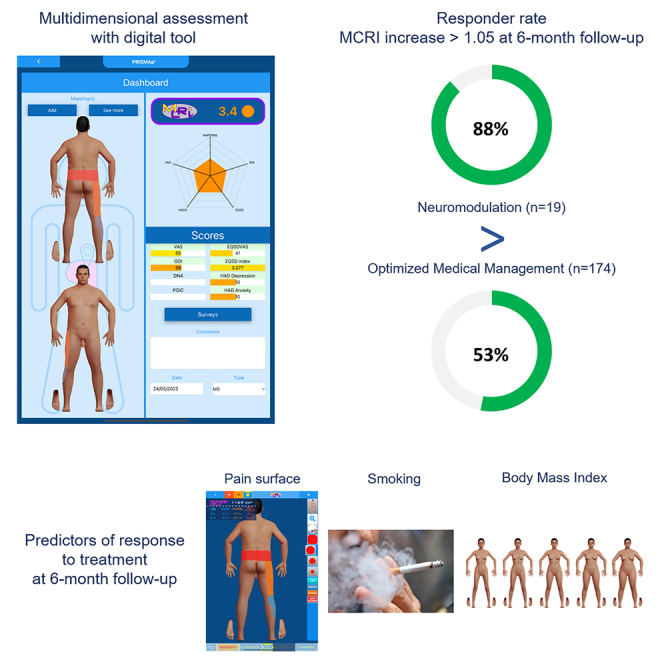

## Introduction

Post-operative refractory pain, named persistent spinal pain syndrome type 2 (PSPS-T2),^1^ represents a global burden disease affecting 10%–40% of operated patients.^2^ The refractory characteristics of post-operative pain result in therapeutic wandering and huge financial cost.^3^ Diagnosis of post-operative refractory pain remains challenging due to the frequent overlap between the mechanical and neuropathic nature of experienced pain.^1^

The first-line treatment of post-operative chronic mechanical pain remains pharmacological (nonsteroidal anti-inflammatory drugs, paracetamol, opioids, etc.), although its efficacy is limited,^4^ and side effects could be dramatic.^5^ Besides, anti-neuropathic pharmacological approach has not shown strong evidence of efficacy.^6^ The limited effects of medication compel clinicians to consider reoperation as a potential option to treat patients with PSPS-T2. However, recent studies have shown that surgical reoperation fails to provide long-term benefits, especially in cases involving neuropathic pain.^7^ Thereby, management of PSPS-T2 represents a challenge reflecting a complex puzzle of daily life impairments that must be remedied so as to optimize a tailored care pathway. Implanted neurostimulation, developed more than 50 years ago, has proven efficacy to manage such neuropathic pain.^8^ The deployment of implanted neurostimulation is still today addressed as a last resort option,^9^ following more than 10 years of medical wandering.^10^ Implanted neuromodulation is never delivered as the sole therapy and always as an adjunction to optimized medical management (OMM). Assessment of neuromodulation combined with OMM is very challenging, and the data collected through randomized controlled trials are conditioned by the technical skills of the implanter, patient selection, and programming expertise.^11^

**Figure 1:**
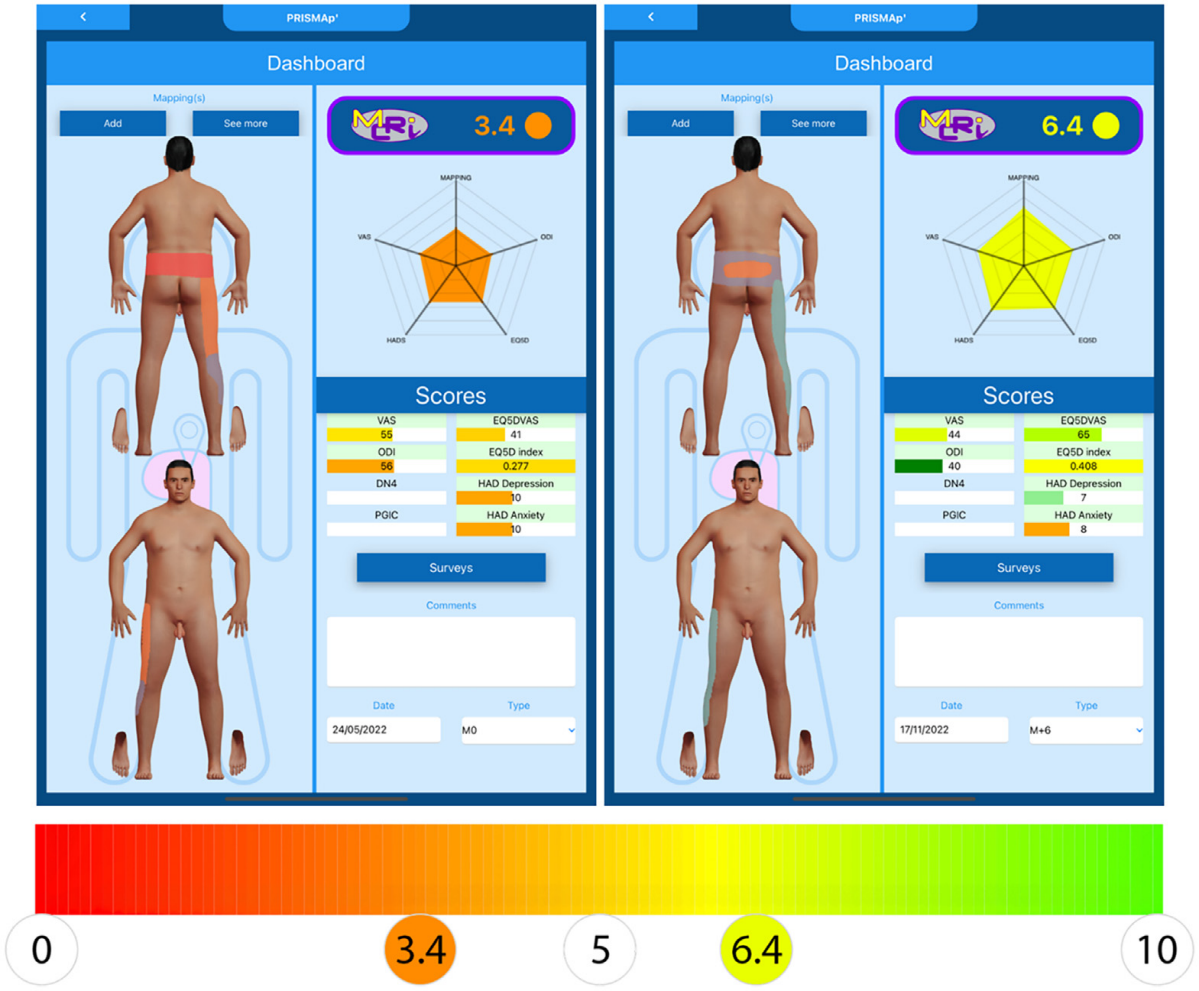
Digital interface used to assess global health state of the patient pre- (left) and post-intervention (right) The MCRI includes pain intensity, functional disability, quality of life, anxiety and depression, and pain surface related to pain intensity. The MCRI is a score that ranges from 0 (worst global health status) to 10 (best global health status).

**Figure 2:**
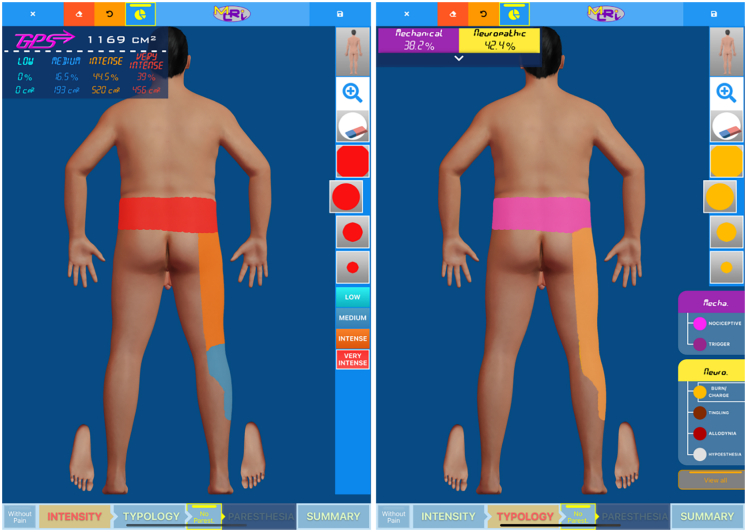
The mapping tool used to assess the global pain surface (GPS) related to pain intensity (left) or pain typology (right)

The only way to assess the relevance of OMM as appropriate treatment in PSPS-T2 with or without surgical reoperation (OMM+resurgery) or neurostimulation (OMM+neuromodulation) is to conduct a prospective real-world investigation, where the patients are assessed using multidimensional index. By using machine learning techniques including 432 variables, we recently created and validated the Multidimensional Clinical Response Index (MCRI).^12^ The MCRI (Figure 1), including pain intensity, pain surface (Figure 2), quality of life, psychological distress, and functional disability components, better reflects the Patient Global Impression of Change (PGIC) than other, one-dimensional pain assessments. The development of comprehensive multidimensional outcomes, encapsulated in an innovative digital tool, would offer new opportunities to predict responsiveness to therapies and identify the best management approach for the vulnerable PSPS-T2 population.

By using a digital medicine tool specifically dedicated to evaluate the global health status of a chronic pain population, we aimed to determine the effectiveness of referral to OMM with or without surgical reoperation (OMM+resurgery) or neurostimulation (OMM+neuromodulation) at 6 months in patients with PSPS-T2 through a real-life prospective multicenter observational trial. We also aimed to determine treatment efficacy at 3-, 6-, 9-, and 12-month follow-up periods with the MCRI and unidimensional assessment, by comparing the different approaches using propensity score analysis. Finally, we aimed to determine an algorithm to identify predictive outcomes for determining patient responders after 6-month follow-up.

## Results

### Patient characteristics

**Figure 3:**
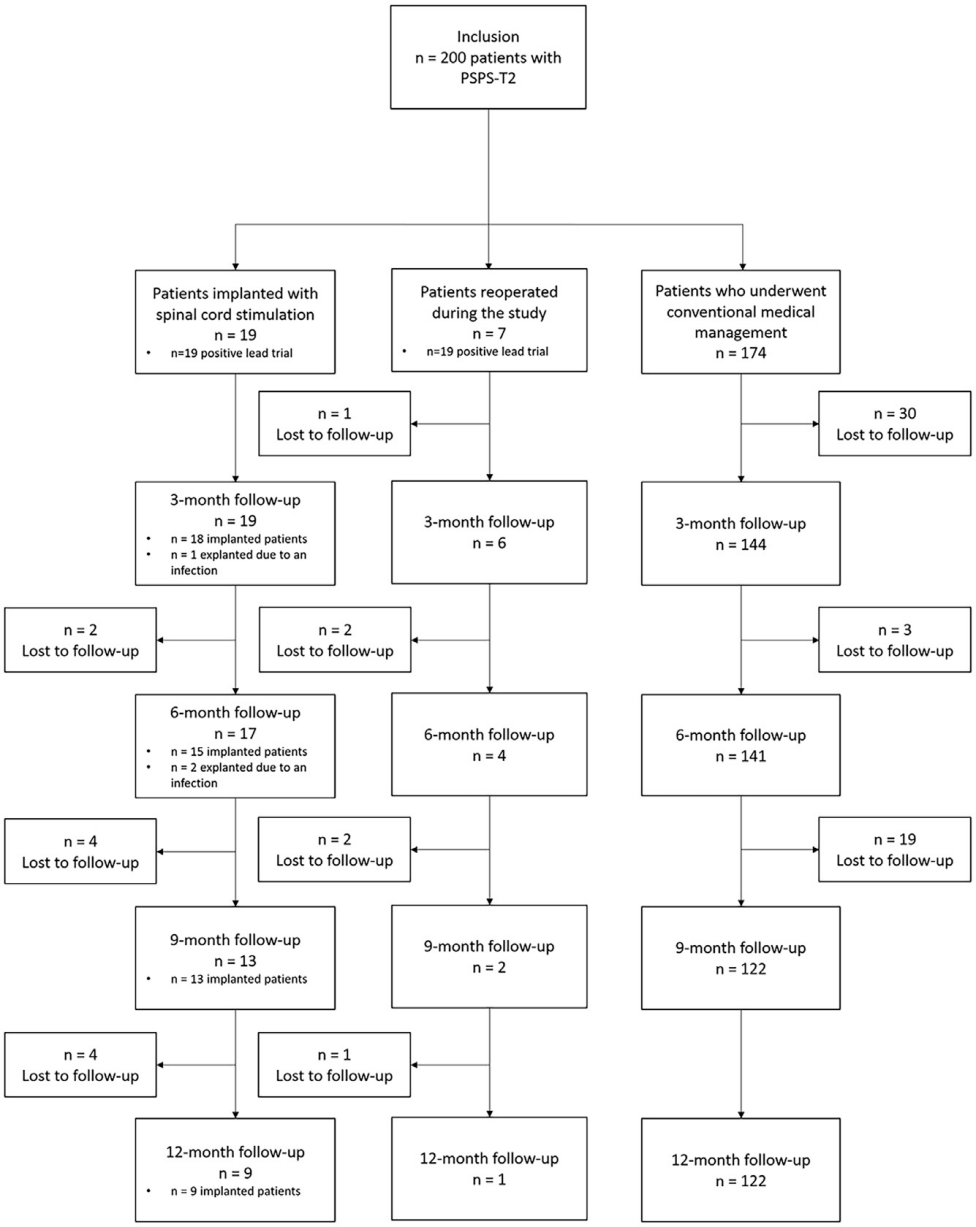
Flowchart of study participants

We included 200 patients: 174 were allocated to the OMM group, 19 to the neurostimulation group, and 7 to the reoperation group (Figure 3). Baseline characteristics of the three groups are presented in Table 1 (STAR Methods). After overlap weighting, all covariate differences between groups were negligible. In the IN group, all patients had a positive lead trial. However, two patients were explanted due to an infection but were followed for 6 months until the end of the study and were included in the primary 6-month follow-up analysis.

**Table 1:** Patients’ baseline characteristics (*n* = 193) for the unweighted groups and the effect sizes of the difference between the two unweighted and weighted groups

Variables Mean (SD)—n (%)	IN (*n* = 19)	OMM (*n* = 174)	Reoperation (*n* = 7)	Unweighted ES^a^ IN vs. OMM	Weighted ES^a^ IN vs. OMM
Age	49.2 (11.1)	53.3 (12.6)	53.1 (16 · 3)	0.353 (M^b^)	<10^−8^ (N)
Gender				0.064 (N)	<10^−8^ (N)
Male	10 (52.6%)	73 (42.0%)	4 (57.1%)		
Female	9 (47.4%)	101 (58.0%)	3 (42.9%)		
BMI	29.1 (6.4)	27.5 (4.9)	29.2 (6.8)	−0.278 (S)	<10^−8^ (N)
Smoking status				0.058 (N)	<10^−8^ (N)
Smoker	9 (47.4%)	66 (37.9%)	5 (71.4%)		
Non-smoker	10 (52.6%)	108 (62.1%)	2 (28.6%)		
Duration of current pain in years (median [min–max])	3 [1–37]	2 [0–31]	2 [1–21]	−0.240 (S)	<10^−8^ (N)
Number of ongoing analgesic medication (median [min–max])	2 [0–6]	2 [0–8]	2 [0–6]	−0.392 (M)	0.045 (N)
Mechanical/neuropathic back pain semiology				0.148 (S)	<10^−8^ (N)
Mechanical	13 (68.4%)	114 (65.5%)	6 (85.7%)		
Neuropathic	3 (15.8%)	9 (5.2%)	0 (0%)		
Mixed	3 (15.8%)	51 (29.3%)	1 (14.3%)		
Mechanical/neuropathic leg pain semiology				0.147 (S)	<10^−8^ (N)
Mechanical	1 (5.3%)	9 (5.2%)	1 (14.2%)		
Neuropathic	14 (73.7%)	118 (67.8%)	3 (42.9%)		
Mixed	1 (5.3%)	34 (19.5%)	3 (42.9%)		
No leg pain	2 (10.5%)	6 (3.4%)	0 (0%)		
Unknown	1 (5.3%)	7 (4.0%)	0 (0%)		
Number of spinal surgeries				0.33 (M)	<10^−8^ (N)
1	6 (31.6%)	92 (52.9%)	3 (42.9%)		
2	7 (36.8%)	48 (27.6%)	4 (57.1%)		
3	2 (10.5%)	26 (14.9%)	0 (0%)		
4	1 (5.3%)	7 (4.0%)	0 (0%)		
5+	3 (15.8%)	1 (0.6%)	0 (0%)		
Type of previous spine surgeries				0.082 (N)	<10^−8^ (N)
Fusion	3 (15.8%)	26 (14.9%)	2 (28.6%)		
Decompression	12 (63.2%)	127 (73.0%)	4 (57.1%)		
Fusion & decompression	4 (21.1%)	21 (12.1%)	1 (14.3%)		
Back/leg pain predominance				0.073 (N)	<10^−4^ (N)
Back	8 (42.1%)	91 (52.3%)	5 (71.4%)		
Leg	11 (57.9%)	81 (46.6%)	2 (28.6%)		
Equivalent	0 (0%)	2 (1.1%)	0 (0%)		
NPRS	6.9 (1.0)	6.0 (1.5)	6.0 (1.2)	−0.741 (M)	−0.095 (N)
EQ-5D index	0.24 (0.18)	0.28 (0.24)	0.26 (0.36)	0.201 (S)	<10^−8^ (N)
ODI	46.5 (11.8)	44.4 (13.4)	58.0 (15.0)	−0.165 (S)	0.069 (N)
HADS depression	9.3 (4.0)	8.5 (3.8)	10.7 (4.0)	−0.196 (S)	<10^−7^ (N)
HADS anxiety	9.5 (3.2)	10.0 (3.9)	10.0 (5.2)	0.150 (S)	<10^−9^ (N)
Pain surface (median [IQR])	458.4 [468.3]	420.8 [380.2]	574.8 [342.3]	−0.250 (S)	<10^−10^ (N)
PCS catastrophizing score	30.2 (9.6)	28.7 (11.3)	27.7 (11.8)	−0.142 (S)	−0.043 (N)

a The effect size index was Cohen’s d for quantitative variables and Cramer’s V for categorical variables.

b S, small effect size; M, moderate effect size; N, negligible effect size. SD, Standard deviation; DN4, Douleur Neuropathique (neuropathic pain) in 4 question; NPRS: Numeric Pain Rating Scale; EQ-5D: EuroQuol-5 dimensions; ODI: Oswestry Disability Index; HADS: Hospital Anxiety and Depression Scale; PCS: Pain Catastrophizing Scale.

### Outcomes

Results were presented first with within-group comparisons for each treatment separately. Secondly, we conducted between-groups comparisons for each outcome. Due to the very small sample size of the reoperation group, the between-treatment effect was only compared between the OMM and neurostimulation groups.

### Primary outcome

No statistically significant difference was observed in the OMM (*p* > 0.07) and Reoperation (*p* > 0.56) groups between MCRI at baseline and follow-up visits, whereas the neurostimulation group exhibited a significant increase in MCRI scores from baseline (3.3 ± 1.5) to 3-month (6.6 ± 2.3, *p* < 0.0001), 6-month (6.9 ± 2.0, *p* < 0.0001), 9-month (6.8 ± 2.3, *p* = 0.0002), and 12-month (6.4 ± 2.4, *p* = 0.008) follow-up (Table 2, STAR Methods).

**Table 2:** Unweighted paired comparisons between baseline and each follow-up for each treatment

	Implanted neurostimulation		Optimized medical management		Reoperation	
	N	N	N	Mean (SD)	N	Mean (SD)
**MCRI/10 (MCID = 1 · 05)**						

Baseline	19	3. 3 (1.5)^a^	174	4.3 (2.1)^a^	7	2.7 (1.6)^a^
3 months	19	6.6 (2.3)^a^	144	4.4 (2.8)^a^	6	4.1 (3.3)^a^
6 months	17	6.9 (2.0)^a^	141	4.4 (3.0)^a^	4	3.2 (3.1)^a^
9 months	13	6.8 (2.3)^a^	122	4.0 (2.9)^a^	2	5.4 (5.2)^a^
12 months	9	6.4 (2.4)^a^	122	3.9 (3.0)^a^	1	0.53 (–)

**NPRS/10 (MCID = 2)**						

Baseline	19	6.9 (1.0)^a^	174	6.0 (1.5)^a^	7	6.0 (1.2)^a^
3 months	19	3.7 (2.2)^a^	144	5.5 (2.1)^a^	6	5.3 (2.0)^a^
6 months	17	3.8 (2.7)^a^	141	5.4 (2.1)^a^	4	6.3 (2.2)^a^
9 months	13	3.3 (2.1)^a^	122	5.3 (2.0)^a^	2	5.5 (3.5)^a^
12 months	9	3.7 (2.1)^a^	122	5.3 (1.9)^a^	1	8 (–)

**ODI/100 (MCID = 10)**						

Baseline	19	46.5 (11.8)^a^	174	44.4 (13.4)^a^	7	58.1 (15.0)^a^
3 months	19	38.5 (17.4)^a^	144	42.2 (14.9)^a^	6	50.0 (15.8)^a^
6 months	17	36.9 (15.9)^a^	141	41.5 (16.2)^a^	4	62.8 (16.6)^a^
9 months	13	34.5 (15.8)^a^	122	41.7 (15.7)^a^	2	37.4 (12.0)^a^
12 months	9	37.3 (18.5)^a^	122	41.0 (15.7)^a^	1	46 (−)

**EQ-5D-5L/1 (MCID = 0**.**19)**						

Baseline	19	0.24 (0.18)^a^	174	0.28 (0.24)^a^	7	0.26 (0.36)^a^
3 months	19	0.36 (0.22)^a^	144	0.32 (0.26)^a^	6	0.29 (0.30)^a^
6 months	17	0.44 (0.20)^a^	141	0.33 (0.28)^a^	4	0.10 (0.37)^a^
9 months	13	0.41 (0.29)^a^	122	0.33 (0.28)^a^	2	0.41 (0.24)^a^
12 months	9	0.40 (0.29)^a^	122	0.33 (0.27)^a^	1	0.24 (−)

**HADS depression/21 (MCID = 2)**						

Baseline	19	9.3 (4.0)^a^	174	8.5 (3.8)^a^	7	10.7 (4.1)^a^
3 months	19	8.7 (4.8)^a^	144	8.0 (4.0)^a^	6	10.3 (4.9)^a^
6 months	17	8.5 (4.7)^a^	141	8.3 (4.2)^a^	4	11.5 (7.0)^a^
9 months	13	8.5 (5.3)^a^	122	8.3 (4.2)^a^	2	5.0 (2.8)^a^
12 months	9	7.7 (4.6)^a^	122	8.0 (4.4)^a^	1	7.0 (−)

**HADS anxiety/21 (MCID = 2)**						

Baseline	19	9.5 (3.2)^a^	174	10.0 (3.9)^a^	7	10.0 (5.4)^a^
3 months	19	8.0 (3.5)^a^	144	9.5 (4.1)^a^	6	9.7 (6.2)^a^
6 months	17	8.1 (3.5)^a^	141	9.3 (4.4)^a^	4	11.8 (4.3)^a^
9 months	13	7.6 (2.9)^a^	122	9.2 (4.3)^a^	2	9.0 (5.7)^a^
12 months	9	8.3 (2.2)^a^	122	8.9 (4.3)^a^	1	13.0 (−)

**Pain surface intensity**^b^**(MCID = 468 cm^2^)**						

Baseline	19	2,247 (1,966)^a^	174	2,028 (2,138)^a^	7	3,004 (2,709)^a^
3 months	19	2,102 (3,223)^a^	144	1,794 (2,339)^a^	6	2,403 (3,214)^a^
6 months	17	1,013 (1,393)^a^	141	1,767 (2,368)^a^	4	2,805 (3,424)^a^
9 months	13	952 (1,357)^a^	122	1,889 (2,727)^a^	2	1,529 (2,089)^a^
12 months	9	1,259 (1,472)^a^	122	1,924 (2,612)^a^	1	3,007 (−)

**PGIC**^c^						

Baseline	–	–	–	–	–	–
3 months	19	5.2 (1.6)^a^	144	3.4 (1.7)^a^	6	3.7 (1.5)^a^
6 months	17	5.3 (1.5)^a^	141	3.5 (1.7)^a^	4	3.0 (2.2)^a^
9 months	13	5.7 (0.9)^a^	122	3.5 (1.8)^a^	2	4.0 (2.8)^a^
12 months	9	5.7 (1.6)^a^	122	3.5 (1.7)^a^	1	2 (−)

MCID, minimal clinically important difference; SD, standard deviation; MCRI, Multidimensional Clinical Response Index; NPRS, Numeric Pain Rating Scale; ODI, Oswestry Disability Index; EQ-5D, EuroQuol-5 dimensions; HADS, Hospital Anxiety and Depression Scale; PGIC, Patient Global Impression of Change.

a Visits with different superscripts are significantly different.

b Pain surface intensity (PSI) is calculated as the sum of the different surfaces (cm^2^) multiplied by their intensity (i.e., PSI = 1∗low pain surface +2∗moderate pain surface +3∗intense pain surface +4∗very intense pain surface).

c Since PGIC already assesses change, the value at follow-up was taken directly instead of difference between baseline and follow-up.

Results showed clinically and statistically higher MCRI for the neurostimulation group compared to the OMM group at 3 months (difference: +2.9, 95% confidence interval [CI] [2.06, 3.65], *p* < 0.0001), 6 months (difference: +2.8, 95% CI [1.97, 3.59], *p* < 0.0001), 9 months (difference: +2.6, 95% CI [1.67, 3.44], *p* < 0.0001) and 12 months (difference: +3.0, 95% CI [1.99, 3.97], *p* < 0.0001) (Table 3, STAR Methods).

**Table 3:** Propensity-weighted comparisons between implanted neurostimulation (IN) and conventional medical treatment (OMM) at each follow-up

	Implanted neurostimulation		Optimized medical treatment				
	N	Mean (SD)	N	Mean (SD)		Mean diff score (CI 95%) OMM versus IN	*p* value of difference
**MCRI/10 (MCID = 1**.**05)**							

Baseline	19	3.4 (2.0)	174		3.2 (2.1)	0.2 [−0.37, 0.70]	0.55
3 months	19	6.8 (2.6)	144		4.0 (3.5)	2.9 [2.06, 3.65]	*p* < 0.0001
6 months	17	7.0 (2.2)	141		4.2 (3.7)	2.8 [1.97, 3.59]	*p* < 0.0001
9 months	13	6.5 (2.9)	122		4.0 (3.7)	2.6 [1.67, 3.44]	*p* < 0.0001
12 months	9	6.6 (3.4)	122		3.6 (3.6)	3.0 [1.99, 3.97]	*p* < 0.0001

**NPRS/10 (MCID = 2)**							

Baseline	19	6.8 (1.2)	174		6.9 (1.2)	−0.1 [−0.42, 0.21]	0.51
3 months	19	3.4 (2.1)	144		5.6 (2.4)	−2.2 [−2.74, −1.56]	*p* < 0.0001
6 months	17	3.5 (3.0)	141		5.6 (2.2)	−2.1 [−2.79, −1.42]	*p* < 0.0001
9 months	13	3.3 (2.0)	122		5.5 (2.1)	−2.2 [−2.78, −1.68]	*p* < 0.0001
12 months	9	3.4 (1.4)	122		5.4 (2.1)	−2.0 [−2.48, 1.45]	*p* < 0.0001

**ODI/100 (MCID = 10)**							

Baseline	19	46.4 (13.6)	174		47.4 (17.8)	−1.05 [−5.3, 3.2]	0.63
3 months	19	35.9 (18.4)	144		42.1 (22.6)	−6.2 [−11.6, −0.8]	0.025
6 months	17	41.0 (20.1)	141		42.5 (23.0)	−1.5 [−7.2, 4.3]	0.62
9 months	13	38.1 (19.8)	122		43.7 (25.3)	−5.5 [−11.7, 0.7]	0.08
12 months	9	39.4 (24.2)	122		40.9 (24.3)	−1.6 [−8.2, 5.0]	0.64

**EQ-5D-5L/1 (MCID = 0**. .**19)**							

Baseline	19	0.22 (0.27)	174		0.22 (0.24)	0.0 [−0.07, 0.07]	0.99
3 months	19	0.37 (0.26)	144		0.33 (0.33)	0.04 [−0.04, 0.11]	0.34
6 months	17	0.41 (0.28)	141		0.35 (0.31)	0.05 [−0.03, 0.13]	0.19
9 months	13	0.38 (0.39)	122		0.34 (0.35)	0.04 [−0.06, 0.14]	0.43
12 months	9	0.43 (0.38)	122		0.36 (0.37)	0.07 [−0.03, 0.17]	0.16

**HADS depression/21 (MCID = 2)**							

Baseline	19	9.5 (4.6)	174		9.5 (3.9)	0.0 [−1.14, 1.14]	0.99
3 months	19	9.0 (5.1)	144		8.1 (4.6)	0.9 [−0.32, 2.25]	0.14
6 months	17	10.1 (6.1)	141		7.5 (4.3)	2.6 [1.20, 3.96]	0.0003
9 months	13	10.4 (6.4)	122		7.7 (5.2)	2.7 [1.15, 4.21]	0.0007
12 months	9	8.4 (6.8)	122		7.3 (5.4)	1.1 [−0.47, 2.77]	0.16

**HADS anxiety/21 (MCID = 2)**							

Baseline	19	10.0 (3.4)	174		10.0 (4.5)	0.0 [−1.07, 1.07]	0.99
3 months	19	8.0 (3.3)	144		9.1 (4.5)	−1.0 [−2.09, −0.005]	0.049
6 months	17	9.5 (4.6)	141		8.0 (4.9)	1.6 [0.30, 2.83]	0.016
9 months	13	8.5 (3.5)	122		8.3 (4.8)	0.2 [−0.99, 1.33]	0.78
12 months	9	8.1 (2.8)	122		8.2 (5.0)	−0.1 [−1.28, 1.10]	0.88

**Pain surface intensity**^a^**(MCID = 468 cm^2^)**							

Baseline	19	2,227 (1,774)	174		2816 (4,091)	−750 [−1,572; 73]	0.07
3 months	19	1,449 (2,159)	144		2,680 (4,948)	−1,230 [−2,228; −233]	0.016
6 months	17	987 (992)	141		1,755 (2,365)	−768 [−1,255; −281]	0.0022
9 months	13	1,278 (1,723)	122		2,311 (3,769)	−1,034 [−1,856; −212]	0.014
12 months	9	1,687 (1,978)	122		2,454 (4,137)	−766 [−1,733; 200]	0.12

**PGIC**^b^							

Baseline	–	–	–		–	–	–
3 months	19	5.6 (1.1)	144		3.3 (2.3)	2.3 [1.8, 2.8]	*p* < 0.0001
6 months	17	5.1 (1.4)	141		3.4 (2.1)	1.7 [1.3, 2.2]	*p* < 0.0001
9 months	13	5.5 (1.0)	122		3.2 (2.1)	2.4 [1.9, 2.8]	*p* < 0.0001
12 months	9	4.9 (2.0)	122		2.7 (1.7)	2.1 [1.6, 2.6]	*p* < 0.0001

∗S, small effect size; M, moderate effect size; N, negligible effect size; SD, standard deviation; MCRI, Multidimensional Clinical Response Index; NPRS, Numeric Pain Rating Scale; ODI, Oswestry Disability Index; EQ-5D, EuroQuol-5 dimensions; HADS, Hospital Anxiety and Depression Scale; PGIC, Patient Global Impression of Change.

a Pain surface intensity (PSI) is calculated as the sum of the different surfaces (cm^2^) multiplied by their intensity (i.e., PSI = 1∗low pain surface + 2∗moderate pain surface + 3∗intense pain surface + 4∗very intense pain surface).

b Since PGIC already assesses change, the value at follow-up was taken directly instead of difference between baseline and follow-up.

Moreover, the propensity-weighted proportion of MCRI responders (≥1.05 increase) was consistently higher in the neurostimulation group compared to the OMM group at all follow-up visits (3 months: 84% versus 48%, *p* < 0.001; 6 months: 88% versus 53%, *p* < 0.001; 9 months: 79% versus 59%, *p* = 0.008; and 12 months: 74% versus 42%, *p* < 0.001).

### Secondary outcomes

We observed statistically significant NPRS, ODI, EQ5D-5L, and pain surface intensity changes from baseline to follow-up visits for the OMM group, without any significant difference for the HADS (Table 2, STAR Methods). Similarly, we showed statistically significant NPRS, ODI, and EQ-5D-5L changes from baseline to follow-up visits for the neurostimulation group, with significant decrease of pain surface intensity from baseline to 6-month follow-up and without any difference for HADS (Table 2, STAR Methods). The reoperation group did not show any statistically significant improvement in the pain intensity, ODI, EQ5D-5L, and pain surface intensity scores.

At baseline, the propensity-weighted means for all secondary outcomes were comparable between the OMM and the neurostimulation (Table 3, STAR Methods). The neurostimulation group consistently reported significantly lower NPRS scores compared to the OMM group for all follow-up visits. The neurostimulation group reported a significantly lower ODI score than the OMM group at 3 months, without any difference at the other follow-up visits. No significant difference of EQ-5D-5L between neurostimulation and OMM groups was observed in any of the follow-up visits. The improvement of the HADS depression score was significantly greater at 6- and 9-month follow-ups for the OMM than the neuromodulation group, without any difference at 3- and 12-month follow-ups. The improvement of the HADS anxiety score was significantly greater at 3 months for the neuromodulation group compared to the OMM group, whereas the improvement was greater for the OMM group at 6 months. No significant difference was observed at 9 and 12 months. The decrease in pain surface was significantly greater for the neuromodulation than the OMM group at all follow-up visits. We observed a more satisfactory PGIC score for neurostimulation group compared to OMM group at all follow-up visits.

The propensity-weighted proportion of NPRS responders was consistently higher in the neurostimulation group than in the OMM group at all follow-up visits (Table 4). The propensity-weighted proportion of ODI responders was higher in the neurostimulation group compared to the OMM group at 3 months, without any difference at the other follow-up visits. No significant difference of the propensity-weighted proportion of EQ-5D-5L and HADS anxiety responders was observed during any of the follow-up visits. The propensity-weighted proportion of HADS depression responders was significantly lower in the neurostimulation compared to the OMM group at 6 months, without any difference at the other follow-up visits. The propensity-weighted proportion of pain surface responders was significantly higher between the neurostimulation and OMM groups at 3 and 12 months, without any difference at 6 and 9 months. The propensity-weighted proportion of PGIC responders was significantly higher for neurostimulation compared to OMM group at 3 and 12 months, without any difference at 6 and 9 months.

**Table 4:** Unweighted and propensity-weighted comparisons of the proportion of responders for the implanted neurostimulation (IN) group and conventional medical treatment (OMM) group at each follow-up

	Unweighted				Weighted		
	Implanted neurostimulation responders		OMM responders		Implanted neurostimulation	OMM	
	N	N responders (%)	N	N responders (%)	%	%	*p* value of difference
**MCRI increase >1**.**05**							

3 months	19	16 (84%)	144	45 (31%)	84%	48%	*p* < 0..001
6 months	17	15 (88%)	141	51 (36%)	88%	53%	*p* < 0.001
9 months	13	10 (77%)	122	42 (34%)	79%	59%	0.0083
12 months	9	7 (78%)	122	41 (34%)	74%	42%	*p* < 0.001

**NPRS decrease ≥2**							

3 months	19	13 (68%)	144	32 (22%)	72%	54%	0.027
6 months	17	14 (82%)	141	43 (30%)	88%	36%	*p* < 0.001
9 months	13	9 (69%)	122	44 (36%)	98%	59%	*p* < 0.001
12 months	9	6 (67%)	122	42 (34%)	98%	59%	*p* < 0.001

**ODI decrease ≥10**							

3 months	19	8 (42%)	144	29 (20%)	54%	34%	0.015
6 months	17	8 (47%)	141	41 (29%)	43%	33%	0.22
9 months	13	7 (54%)	122	30 (25%)	50%	35%	0.09
12 months	9	4 (44%)	122	47 (39%)	49%	48%	0.95

**EQ-5D-5L increase ≥0**.**19**							

3 months	19	7 (37%)	144	31 (22%)	35%	37%	0.81
6 months	17	9 (53%)	141	32 (23%)	51%	44%	0.37
9 months	13	6 (46%)	122	25 (20%)	43%	32%	0.18
12 months	9	4 (44%)	122	34 (28%)	49%	54%	0.48

**HADS depression decrease ≥2**							

3 months	19	8 (42%)	144	46 (32%)	37%	36%	0.90
6 months	17	9 (53%)	141	48 (34%)	44%	63%	0.02
9 months	13	7 (54%)	122	37 (30%)	39%	49%	0.27
12 months	9	5 (56%)	122	44 (36%)	59%	56%	0.70

**HADS anxiety decrease ≥2**							

3 months	19	12 (63%)	144	55 (38%)	69%	59%	0.17
6 months	17	10 (59%)	141	58 (41%)	47%	60%	0.12
9 months	13	8 (62%)	122	54 (44%)	57%	54%	0.69
12 months	9	5 (56%)	122	54 (44%)	58%	65%	0.47

**Pain surface intensity decrease >468 cm^2^ or decrease by 30%**							

3 months	19	15 (79%)	144	65 (45%)	77%	44%	0.0013
6 months	17	14 (82%)	141	78 (55%)	73%	83%	0.67
9 months	13	9 (69%)	122	59 (48%)	73%	69%	0.38
12 months	9	7 (78%)	122	61 (50%)	76%	57%	0.03

**PGIC >5**							

3 months	19	10 (53%)	144	20 (14%)	59%	21%	*p* < 0.001
6 months	17	8 (47%)	141	23 (16%)	44%	33%	0.15
9 months	13	5 (38%)	122	23 (19%)	31%	22%	0.60
12 months	9	6 (67%)	122	23 (19%)	42%	17%	0.027

MCRI, Multidimensional Clinical Response Index; NPRS, Numeric Pain Rating Scale; ODI, Oswestry Disability Index; EQ-5D, EuroQuol-5 dimensions; HADS, Hospital Anxiety and Depression Scale; PGIC, Patient Global Impression of Change.

### Predictors of response after 6-month follow-up

In the multivariate analysis for predicting response to the assigned treatment, after adjusting for baseline MCRI and treatment, we found that tobacco smoking, higher BMI, larger pain surface, and OMM therapy were associated with a lower likelihood of response (Table 5). Considering a significance level at 10%, older patients and patients with a lower baseline EQ-5D score were less likely to respond. The model had 10-fold cross-validation area under than curve (AUC) of 0.729 with a standard error of 0.060.

**Table 5:** Univariate and best subset selection analysis of factors associated with outcome

Variables Mean (SD)—n (%)	6-month responders (*n* = 66)	6-month non-responders (*n* = 92)	Univariate *p* value	Standardized odds ratio and 95% CI	Adjusted *p* value
Therapy Implanted neurostimulation OMM	15 (22.7%) 51 (77.3%)	2 (2.2%) 90 (97.8%)	0.00012	0.14 [0.01; 0.81]	0.041
Baseline MCRI	3.8 ± 1.8	4.6 ± 2.2	0.039	0.08 [0.004; 0.66]	0.029
NPRS	6.5 ± 1.2	5.8 ± 1.6	0.0054	–	–
EQ-5D index	0.29 ± 0..20	0.28 ± 0.25	0.51	1..76 [0.94; 3.45]	0.085
ODI	45.4 ± 11.6	43.2 ± 13.0	0.38	–	–
HADS depression	9.2 ± 3.9	7.9 ± 3.3	0.040	–	–
HADS anxiety	9.8 ± 3.9	9.9 ± 3.7	0.58	–	–
Pain surface (median [IQR])	472.8 ± 316.8	525.9 ± 430.3	0.63	0.47 [0.24; 0.83]	0.018
PCS catastrophizing score	30.8 ± 9.9	27.4 ± 11.3	0.055	–	–
Age	51.9 ± 12.0	53.7 ± 11.7	0.18	0.66 [0.40; 1.04]	0.081
Gender			0.22	–	–
Male	25 (37.9%)	43 (46.7%)		–	–
Female	41 (62.1%)	49 (53.3%)		–	–
BMI	27.5 ± 5.3	28.2 ± 4.9	0.27	0.62 [0.38; 0.98]	0.045
Smoking status			0.51	0.61 [0.38; 0.95]	0.035
Smoker	23 (34.8%)	38 (41.3%)		–	–
Non-smoker	43 (65.2%)	54 (58.7%)		–	–
Duration of current pain in years (median [min-max])	3 [0–37]	2 [0–31]	0.20	–	–
Number of ongoing analgesic medication (median [min-max])	2 [0–6]	1.5 [0–6]	0.73	–	–
Mechanical/neuropathic back pain			0.85	–	–
Mechanical	43 (65.2%)	60 (65.2%)		–	–
Neuropathic	3 (4.5%)	6 (6.5%)		–	–
Mixed	20 (30.3%)	26 (28.3%)		–	–
Mechanical/neuropathic leg pain			0.71	–	–
Mechanical	3 (4.5%)	5 (5.4%)		–	–
Neuropathic	45 (68.2%)	65 (70.7%)		–	–
Mixed	13 (19.7%)	17 (18.5%)		–	–
No leg pain	1 (1.5%)	3 (3.3%)		–	–
Unknown	4 (6.1%)	2 (2.2%)		–	–
Number of spinal surgeries			0.96	–	–
1	33 (50.0%)	51 (55.4%)		–	–
2	19 (28.8%)	24 (26.1%)		–	–
3	9 (13.6%)	12 (13.0%)		–	–
4	3 (4.5%)	3 (3.3%)		–	–
5+	2 (3.0%)	2 (2.2%)		–	–
Type of previous spine surgeries			0.24	–	–
Fusion	12 (18.2%)	10 (10.9%)		1.00 [0.22; 4.15]	0.99
Decompression	50 (75.8%)	71 (77.2%)		–	–
Fusion & decompression	4 (6.1%)	11 (12.0%)		0.38 [0.08; 1.59]	0.21
Back/leg pain predominance			0.67	–	–
Back	34 (51.5%)	43 (46.7%)		–	–
Leg	32 (48..5%)	49 (53.3%)		–	–
Equivalent	0 (0%)	0 (0%)		–	–
Baseline MCRI × Therapy = OMM^a^	4.2 ± 1.8	4.7 ± 2.2	0.02	4.26 [0.53; 72.56]	0.20
Baseline MCRI × Therapy = IN^b^	3.0 ± 1.6	4.8 ± 1.6	–	–	–

OMM, Optimized Medical Management; MCRI, Multidimensional Clinical Response Index; NPRS, Numeric Pain Rating Scale; EQ-5D, EuroQuol-5 Dimensions; ODI, Oswestry Disability Index; HADS, Hospital Anxiety and Depression Scale; PCS, Pain Catastrophizing Score; BMI, Body Mass Index.

In the multivariate analysis, only variables selected with the best subset selection algorithm were shown.

a Mean baseline MCRI for responders and non-responders in the OMM group.

b Mean baseline MCRI for responders and non-responders in the implanted neurostimulation group.

Additionally, an interaction term between baseline MCRI and therapy groups was incorporated into the model to assess potential differences in the effect of baseline MCRI on clinical outcomes between the OMM and implanted neurostimulation groups. This interaction effect did not reach statistical significance (Table 5).

## Discussion

In this prospective observational real-life study involving 200 PSPS-T2 patients with 12-month follow-up, multidimensional assessment showed that neurostimulation was significantly effective over time, whereas OMM and reoperation were not. In addition, using propensity score, neurostimulation showed greater clinical benefits than OMM treatment. Finally, our predictive model highlighted that pain surface, BMI, and smoking status are significant predictors of outcomes.

### Pathway and treatment efficacy

#### Re-operation

The literature reported that after one or several fusion(s), the success rate of a new spinal surgery remains very low, if not inexistent.^13^ Investigating 182 revision surgeries in patients with PSPS-T2, Fritsch et al.^14^ showed that recurrence of disc herniation decreases from 42% in patients with one revision surgery to 0% in patients with four or more reoperations. Although spinal re-surgery cannot be indicated for all patients with PSPS-T2, surgery should not be ruled out before a meticulous, systematic, and complete work-up, aimed at identifying the potential source of residual mechanical pain.^15^ Surgical anatomical restoration, even adequately performed, cannot be decently extrapolated to clinical success in a specific PSPS-T2 population presenting mainly with neuropathic or mixed neuropathic/mechanical pain.

#### OMM: An optimized pathway?

In first-line treatment, due to low efficacy, poorer long-term outcomes,^4^ and substantial risk exposure to cardiovascular disease, cognitive impairment, and addiction, optimal medication management is nowadays recommended only in case of inadequate response to non-pharmacological interventions,^9^ Having combined non-pharmacological and pharmacological treatments, the OMM group failed to report significant outcome improvement over the 12-month follow-up. To date, OMM has occasioned no consensus and has shown extreme variability depending on practices, convictions, cultures, health systems, and countries. These barriers could be overcome by replicating the current prospective cohort design on a larger scale via registries to collect more data and reflect the originality of specific practices by data mining.

#### Neurostimulation: Technological advances and patient selection

Neurostimulation is currently recommended as a last-resort option to manage refractory neuropathic pain.^9^ Technological innovation has markedly evolved, delivering stimulation with different types of temporal and spatial neural targeting.^10^^,^^16^ In a recent randomized crossover trial investigating SCS efficacy between paresthesia-based and paresthesia-free stimulations, we reported that a device with versatile capability improved responder rate by up to 25%.^17^

On the other hand, patient selection represents a pivotal point requiring specific focus to predict positive or negative outcomes of implanted neurostimulation.^18^ With an implanted neurostimulation eligibility rate of 46%, Turner et al.^19^ reported a success rate of 6% with spinal cord stimulation, showing no difference in clinical outcomes between neurostimulation and usual care. Although our eligibility rate could be perceived as restrictive (9.5%), it may be the price to pay to achieve clinical benefits (88% at 6 months). Recently, Goudman et al.^18^ proposed a sequential decision-making model including initial indication, psychological factors, goal-setting, ethical issues, and predictive approach. By continuing to advance in technology and increasingly refined patient selection, implanted neurostimulation might be considered as an option in the right clinical hand, for the right patient, at the right moment.

### Holistic assessment and digital medicine

The primary objective in incorporating the MCRI into a digital tool for evaluating treatment effectiveness was to proactively move away from relying solely on NRPS or VAS, which may not inherently encapsulate the full spectrum of patient needs and expectations. Multidimensional assessment better captures patients’ global health than unidimensional scoring. This is demonstrated by the stronger correlation of MCRI with various dimensions of pain compared to unidimensional assessment.^12^ Furthermore, our findings indicated that MCRI exhibited heightened sensitivity and specificity in relation to the Patient Global Impression of Change (PGIC) compared to unidimensional outcomes.^12^

The digital mapping tool can provide, for the very first time, objective quantification of pain surface related to pain intensity, subsequently delineating the distribution of mechanical and neuropathic pain components (see Figure 2). In addition, mechanical pain can be subdivided into nociceptive or trigger-related, whereas neuropathic pain can be compartmentalized into four distinct types: burn/charge, tingling, allodynia, and hypoesthesia. Investigating pain using an objective tool combined with questionnaires represents a significant breakthrough in pain assessment. Utilization of this digital tool within the PSPS-T2 population, as part of our study, offers the potential to assist clinicians in refining pain assessment in a more general painful population.

Predictive models implemented in digital tools represent the future for decision-making in view of optimizing the patient care pathway. Using multivariate analysis, we found that baseline MCRI was associated with patient outcome. We observed that patients with a lower baseline MCRI were more likely to respond positively to treatment. This finding suggests that it may be easier to improve a low MCRI by enhancing one or more dimensions over the course of therapy, whereas a higher baseline MCRI may be more resistant to change. Additionally, we found a significant association between the type of therapy and clinical outcomes, with implanted neurostimulation yielding superior response rates compared to OMM. These results should be interpreted with caution for two key reasons. First, there was a notable imbalance in group sizes between the OMM and implanted neurostimulation groups (22.7% and 77.3%, respectively), which may introduce bias. Second, as previously mentioned, the selection of patients for implanted neurostimulation was highly selective, as is typical in clinical practice, which could contribute to confounding bias. We mitigated this bias by employing propensity score methods for the main comparisons. These factors may also explain the non-significant interaction effect. To further refine the predictive model, a randomized controlled trial involving patients eligible for implanted neurostimulation, or a larger cohort exclusively composed of patients receiving implanted neurostimulation, would be beneficial.

Our predictive model also revealed that pain surface, BMI, and smoking status are significant predictors of outcomes, and EQ-5D-5L as well as age, to a lesser extent, with AUC of 0.728. In their model applied in 1965 low back pain patients, Khor et al.^20^ reported that smoking status and previous spinal surgery were both predictive outcomes of spinal surgery, with AUC of 0.66–0.79. Previous studies investigating predictive response to implanted neuromodulation in patients with PSPS-T2 showed that age, duration of pain, depression, medication use, and higher heart rate were significant outcome predictors,^21^^,^^22^ with AUC of 0.79–0.83. Ultimately, this advancement could pave the way for predicting therapeutic responders in the near future with big data and data mining.^21^

### Limitations of study

OMM is not an object of current consensus, and there is extreme variability depending on practices, convictions, cultures, health systems, countries, and center/institution. This heterogeneity underscores the importance of multidimensional assessment including pain mapping through a digital medicine tool, thereby possibly avoiding misinterpretation of pain physiopathology and typology. Capturing neurostimulation effect can be challenging in observational study due to the many possibilities afforded by the different anatomical targets (i.e., spinal cord, ganglion, peripheral nerve, subcutaneous), as well as diversified waveforms and electric current steering. The fact that our study was not a randomized controlled trial constitutes a major limitation, conclusively indicating that SCS achieved superior results in carefully selected patients and that its results cannot be extended to the entire PSPS-T2 population. Although we used a propensity score approach to capture real-life healthcare management, the sample sizes was unevenly distributed according to treatments, with most patients receiving a conservative approach and a very limited number of patients reoperation and neuromodulation. Thereby, big data analyses through massive registry would be the best way to extrapolate therapy efficacy on a larger scale.

### Conclusion

By assessing the effectiveness of therapy with a composite index exploring health state in multidimensions (MCRI), our study showed that OMM and reoperation did not effectively provide clinical benefits, whereas the patients involved in the neurostimulation pathway did. In addition, smoking has been identified as a negative predictive responder outcome, while less pain surface as positive predictive responder outcome for patient with PSPS-T2. Through recent developments in machine learning technique, digital medicine will strongly contribute to medical decisions rationalizing complex patient pathways, in which today’s problem-solving equations remain challenging or poorly addressed by traditional randomized controlled studies. Future predictive research should be conducted by means of prospective collection of big data through registries, leading to personalization and customization of patient care.

## Resource availability

### Lead contact

Further information and requests should be directed to the lead contact, Dr Maxime Billot (maxime.billot@chu-poitiers.fr).

### Materials availability

This study did not generate new unique reagents.

### Data and code availability

• Data reported in this paper will be shared by the lead contact upon request.
• This paper does not report original code.
• Any additional information required to reanalyze the data reported in this paper is available from the lead contact upon request.

## Acknowledgments

We thank Jeffrey Arsham for his proofreading of the manuscript and his suggestions regarding medical writing.

Fundings: the study was funded by Medtronic (ERP NM-3351).

Trial Registration ClinicalTrials.gov Identifier: NCT02964130: https://clinicaltrials.gov/study/NCT02964130?cond=NCT02964130&rank=1.

## Author contributions

The Trial Steering Committee consisting of P.R. and M.R. designed the study, approved the analysis plan, provided study oversight, and contributed to interpretation of the data; N.N., P.P., B.B., B.L., S.B., G.B.M., and B.R.M. conducted the study. M.B. and A.O. drafted the initial article with input and critical review from L.G., M.M., R.D., and P.R. Statistical analysis was performed by A.O. All authors have read and agreed to the published version of the manuscript.

## Declaration of interests

Philippe Rigoard reports grants and speaker fees from Medtronic, Abbott, and Boston Scientific. Philippe Rigoard is a co-inventor on Patent No. 9986946, held by the CHU of Poitiers, which details a novel method for pain mapping. Nicolas Naïditch reports non-financial support and speaker fees from Medtronic. Philippe Page reports non-financial support from Medtronic. Maarten Moens reports speaker fees from Medtronic and Nevro. All other authors declare no competing interests.

## STAR★Methods

### Key resources table

**Table:** 

REAGENT or RESOURCE	SOURCE	IDENTIFIER
**Software and algorithms**		

PRISMap Version 2024.9.0	Rigoard et al.^23^	https://doi.org/10.1016/j.neuchi.2014.09.003
MCRI algorithm	Rigoard et al.^12^	https://doi.org/10.3390/jcm10214910
R software V4.2.2	R Foundation for Statistical Computing, Vienna, Austria	https://www.r-project.org/

### Experimental model and study participant details

#### Study design and ethics

We used data from the prospective observational multicenter PREDIBACK study (ClinicalTrials.gov NCT02964130). Patients were included in five French expert pain centers (Angoulême, Bressuire, La Rochelle, Niort and Poitiers), each of them bringing together a group of pain physicians, spine surgeons and implanters. Patient recruitment started in January 2017 and ended in March 2018. The study complied with the Declaration of Helsinki and was approved by an Ethics Committee (CCP Ouest III) and the ANSM (2016-A01144-47). Informed written consent was obtained from all patients before enrollment.

#### Study population

Patient with PSPS-T2 who met the following criteria were included in the study: patient had at least one spinal surgery, post-operative leg and/or low back pain for at least six months, and an average global pain score ≥4 as measured by the Numeric Pain Rating Scale (NPRS, ranging from 0 to 10) in the week before inclusion.

Patients with one or several of the following criteria were excluded from the study: has been treated with spinal cord, subcutaneous or peripheral nerve stimulation or an intrathecal drug delivery system; has life expectancy of less than 12 months beyond study enrollment; is unable to undergo study assessments; and investigator suspects substance abuse, based on clinical judgment, which might confound the study results.

### Method details

#### Outcomes

Outcome measures were collected using a digital tool at baseline, and at 3-, 6-, 9- and 12-month follow-up periods. The primary outcome represents overall pain-related health status from the MCRI.^12^ The MCRI includes five weighted dimensions: pain intensity assessed with the Numeric Pain Rating Scale (NPRS), pain surface related to pain intensity assessed with a patented pain mapping tool (PRISMap, Poitiers University Hospital, Poitiers),^23^ psychological distress assessed with Hospital Anxiety and Depression Scale (HADS), functional disability with Oswestry Disability Index (ODI), and quality of life assessed with EuroQuol 5 Dimension questionnaire five level (EQ-5D-5L). The MCRI score ranges from 0 indicating the worst possible global health status, to 10 indicating the best potential global health status. The Minimal Clinically Important Difference (MCID) of the MCRI was established at 1.05 (Figure 1),^12^ and represents a clinically significant improvement of the global health status of a given person.

The secondary endpoints were the mean scores and the proportion of responders at baseline and follow-up visits (3, 6, 9 and 12 months) of the NPRS, ODI, EQ-5D-5L, HADS, Patient Global Impression of Change (PGIC) and pain surface (cm^2^). To assess pain surface, patients were asked to draw their pain surface on a computerized tactile interface in a predetermined body related to body mass index.^23^ A color code was used to assess the pain intensity combined with pain surface: light blue = mild pain, dark blue = moderate pain, orange = intense pain, red = very intense pain (Figure 2).

#### Patient group allocations

Patients were followed according to standard care, where all the patients have access to the OMM pathway. After data extraction, patients were allocated to OMM only, OMM+reoperation or OMM+neurostimulation group. Patients were allocated/referred to different therapies based on real-life practices and clinical recommendations as follows:

All patients underwent OMM therapies. In addition, patients were evaluated to assess the eligibility for re-operation or neurostimulation though a multidisciplinary consultation, including a panel of health care professionals (neurosurgeon, nurse, pain therapist, orthopedist, physiotherapist and psychologist/psychiatrist). Patient’s medical records, which includes medical history, the nature of their illness, previous treatments they have undergone, and their responses to these treatments are first screened during the consultation to assess whether reoperation or neurostimulation implantation could be appropriate. Then, a more comprehensive multidisciplinary evaluation is carried out to evaluate the patient’s condition, their neurological status, the potential risks and benefits of the procedure, and their potential to respond positively to the treatment based on their pain diagnosis, typology, morphology, pain mapping, imaging data, presence of psychological/psychiatric disorders, expectations and goals, social factors, ability to understand and consent to the procedure, and ability to cope with the changes that the treatment might bring. A rigorous selection was performed to select only candidates eligible for reoperation or neuromodulation. Patients selected for spinal reoperation (i.e., arthrodesis, fusion, laminectomy, etc.) followed the routine care pathway. Candidates for neurostimulation underwent a 7-day lead trial with a temporary neurostimulator to assess the potential benefit of neurostimulation on pain relief and daily life activity following the French Guidelines from National Health authorities. Following the trial period, the medical team and the patient make a final common decision about whether to proceed with permanent neurostimulation (if at least 50% pain relief or an impactful improvement in global health are achieved). In this study, the OMM+neurostimulation group includes all candidates having undergone a lead trial, even those who were not permanently implanted due to a negative lead trial or complications (e.g., infections). We have chosen to include the patients who have been explanted in the OMM+neurostimulation group in order to estimate the effectiveness of these therapies in an ITT manner (i.e., all patients who were allocated to neuromodulation were included in the analysis, regardless of wether they got permanently implanted or not). This would allow us to reduce the bias advantaging the neurostimulation therapy due to removing patients with a negative outcome after being referred to neuromodulation.

### Quantification and statistical analysis

#### Descriptive analysis

Quantitative characteristics were described using means or medians depending on the distribution of the variable. Qualitative variables were described using numbers and percentages.

#### Treatment efficacy – Time-effect

Time-effect of different treatment modalities was assessed by comparing outcomes at baseline vs. 3, 6, 9 and 12-month follow-up visits using paired t-test or Wilcoxon test depending on the distribution of the difference between the two visits. Normality was evaluated using a Shapiro-Wilk test. A Bonferroni correction was applied to correct for multiple comparisons.

#### Comparison between treatments

To reduce the bias of non-randomized allocation, we used the covariate balancing propensity score overlap-weighting method^24^ (R software package: PSweight). This method estimates the treatment effect on the overlap (ATO) population, who are patients with a high probability of receiving both treatments. This method was shown to achieve better optimal matching compared to other weighting methods^24^ such as inverse probability weighting and propensity score target weighting.

We balanced for the following potential confounding factors in the logistic regression model: sex, age, pain duration, neuropathic/mechanical pain component evaluation, number of spine surgeries prior to inclusion, baseline outcomes (NPRS, HADS, EQ-5D, ODI, pain surface), number of ongoing medications, smoking, and catastrophizing score. We calculated the propensity score for each patient (the fitted probability of patients receiving the treatment given their confounding characteristics). We evaluated balance using Cohen’s d effect size (ES < 0.1 to be negligible, <0.3 to be small, <0.8 to be moderate and >0.8 to be large).^25^ Although commonly used in large-scale studies, propensity score weighting methods are effective in small sample studies.^26^

All analyses were conducted with R software. P-values below 0.05 were considered significant, excepted for the time effects which were corrected for multiple comparisons. When data from the visit preceding treatment were missing, data from the previous visit were used as baseline. Data that were still missing after these data processing steps were not imputed.

#### Predictors of outcome

This analysis aimed to identify baseline factors associated with the 6-month follow-up outcome of the MCRI (≥1.05 change). Multivariate analysis included all baseline variables, adjusting for treatment and baseline MCRI score. The best subset selection method with the *bestglm* R function with the Morgan Tatar search algorithm^27^ was used to identify the variables yielding the best model fit based on the Akaiki Information Criterion (AIC). The model’s discrimination ability was assessed using the Area Under ROC Curve (AUC) statistic. We reported standardized odds ratios and 95% confidence intervals (95%CI) of the retained logistic regression model.

### ADDITIONAL RESOURCES

Trial Registration ClinicalTrials.gov Identifier: NCT02964130: https://clinicaltrials.gov/study/NCT02964130?cond=NCT02964130&rank=1.
